# Dental Restorative Digital Workflow: Digital Smile Design from Aesthetic to Function

**DOI:** 10.3390/dj7020030

**Published:** 2019-03-28

**Authors:** Gabriele Cervino, Luca Fiorillo, Alina Vladimirovna Arzukanyan, Gianrico Spagnuolo, Marco Cicciù

**Affiliations:** 1 Department of Biomedical and Dental Sciences and Morphological and Functional Imaging, Messina University, 98100 Messina, Italy; gcervino@unime.it (G.C.); lucafiorillo@live.it (L.F.); 2 Institute of Dentistry, I. M. Sechenov First Moscow State Medical University, Moscow 119146, Russia; aav0218@mail.ru (A.V.A.); gianrico.spagnuolo@gmail.com (G.S.); 3 Department of Neurosciences, Reproductive and Odontostomatological Sciences, University of Naples “Federico II”, 80131 Napoli, Italy

**Keywords:** Digital Smile Design, restorative dentistry, dentistry software, dentistry design

## Abstract

Breakthroughs in technology have not been possible without influencing the medical sciences. Dentistry and dental materials have been fully involved in the technological and information technology evolution, so much so that they have revolutionized dental techniques. In this study, we want to create the first collection of articles on the use of digital techniques and software, such as Digital Smile Design. The aim is to collect all of the results regarding the use of this software, and to highlight the fields of use. Twenty-four articles have been included in the review, and the latter describes the use of Digital Smile Design and, in particular, the field of use. The study intends to be present which dental fields use “digitization”. Progress in this field is constant, and will be of increasing interest to dentistry by proposing a speed of treatment planning and a reliability of results. The digital workflow allows for rehabilitations that are reliable both from an aesthetic and functional point of view, as demonstrated in the review. From this study, the current field of use of Digital Smile Design techniques in the various branches of medicine and dentistry have emerged, as well as information about its reliability.

## 1. Introduction

In recent years, prosthetic and implant-prosthetic rehabilitation in the dental field have undergone a strong development in aesthetics and cosmetics, benefiting from both the improvement of some laboratory techniques and the definition of some anatomical criteria useful to the aesthetics of the smile. One of the most noteworthy innovations in the field of prosthetics is undoubtedly represented by the advent of computer-aided design and computer-aided manufacturing (CAD/CAM) technology, which allows professionals to guarantee repeatable and remarkable results from an anatomical–functional point of view. The accessible costs, the small size of the machines, and the relative ease of use make this method passable, even in small-scale outpatient facilities. New processing software, for example, aims to make the entire rehabilitative work-flow digital, simplifying the professional’s work and also facilitating communication with the patient. A quick search on the main informatics engine indicates all of the software available on the market—among them, an important role is undoubtedly played by the Digital Smile Design method. In this work, we will take into consideration all of the articles in the literature regarding the use of this method. This software allows for excellent communication with our patients on the one hand, but on the other, offers the clinician a tool to make the correct therapeutic choice through algorithms. Rehabilitation follows a digital pathway, and the patient can see the result even before starting. These methods allow for accurate planning and guarantee aesthetic, functional, and predictable results [1,2,3]. The software in the medical field, 3D technology, and the bioengineering field have been working in synergy in recent years in order to produce excellent therapeutic tools. The possibility of testing three-dimensional structures virtually, before being able to build or test them on patients, is already a huge step forward; this is possible with finite element analyses, for example, with which you can test useful materials for different dental fields, from fixtures to prosthetics, to the simulation of dental movements [4,5]. Digital Smile Design allows for a thorough workflow simulating the rehabilitation of a patient, simply starting with appropriately calibrated photos. The facial study is usually done using the reference lines, from which the standardized parameters have been developed for the frontal and profile views of the face. The horizontal reference lines used in the frontal view include the interpupillary and intercommissural lines, which provide an overall sense of harmony and horizontal perspective, present in an aesthetically pleasing face. However, the main limitation of this kind of therapeutic method is related to the several anatomical features involved in the rehabilitation. The treatment for giving an “aesthetic smile” to patients is related to the different anatomical areas involved in the treatments, like the teeth, gingiva, mucosa, lip, skin, and so on, which rely on symmetry, shape, and golden proportions. The purpose of this study is to evaluate the effective use of Digital Smile Design techniques in dentistry and other medical fields. These techniques are used in different medical fields, and we have analyzed and categorized all of these fields, and have evaluated the reliability and predictability of these digital techniques.

## 2. Material and Methods

### 2.1. Protocol and Registration

This review is registered at PROSPERO with ID number 122744. PROSPERO is an international database of prospectively registered systematic reviews in health and social care, welfare, public health, education, crime, justice, and international development, where there is a health-related outcome. 

### 2.2. Focus Question

The following focus questions were developed according to the population, intervention, comparison, and outcome (PICO) study design [6]:

- What are the fields of use of the Digital Smile Design software?
- Is Digital Smile Design bringing improvements in the comfort of patients and in their treatments?

### 2.3. Information Sources

The search strategy incorporated examinations of electronic databases, supplemented by hand searches. We searched PubMed, Dentistry, and Oral Sciences Source for relevant studies published in English. A hand search of the reference lists in the articles retrieved was carried out in order to source additional relevant publications and to improve on the sensitivity of the search.

### 2.4. Search

The keywords used in the search of the selected electronic databases included the following: 

● “Digital Smile Design”

The choice of keywords was intended to collect and to record as much relevant data as possible, without relying on electronic means alone to refine the search results.

### 2.5. Selection of Studies

Two independent reviewers singularly analyzed the obtaining papers in order to select the inclusion and exclusion criteria as follows. For the stage of reviewing full-text articles, a complete independent dual revision was performed.

### 2.6. Types of Selected Manuscripts

The review included studies on humans and animal published in English. Letters, editorials, and PhD theses were excluded. 

### 2.7. Types of Studies

The review included all human prospective and retrospective follow-up studies and clinical trials, cohort studies, case-control studies, case series studies, animal studies, and literature reviews published on using Digital Smile Design for rehabilitation and restorative dentistry.

### 2.8. Inclusion and Exclusion Criteria

The full texts of all of the studies of possible relevance were obtained for assessment against the following inclusion criteria:

- Digital Smile Design use for restorative dentistry
- Advantages of Digital Smile Design.

The applied exclusion criteria for the studies were as follows:

- Studies involving patients with other specific diseases, immunologic disorders, or other oral risk-related systemic conditions
- Not enough information regarding the selected topic
- No access to the title and the abstract in English.

### 2.9. Digital Dentistryn

#### Digital Smile Design

Digital Smile Design is a method that allows us to digitally design the smile of our patients, by obtaining a simulation and pre-visualization of the therapeutic result. Patients are often found by the dentist and are immediately subjected to dental services or therapies, without the dentist himself having planned well or having shared the therapeutic project of a tailor-made smile for the patient with them. On the one hand, Digital Smile Design allows the patient to have awareness from the beginning of the therapeutic plan and for them be the first interpreter in the aesthetic and functional rehabilitation of their mouth, and on the other hand, it allows the specialist to tune in better to the expectations and needs of the patient, in order to pursue their shared goals. These protocols therefore allow for a previsualization of the clinical case and of the therapeutic result, and for presenting the patient, in a clear way, the usefulness of being able to program the rehabilitation and interface clearly with the help of other professional figures. Being able to provide all of the data to the dental technician, or even being able to evaluate the prosthetic–implant–orthodontic rehabilitation is made simpler, by being able to communicate information about the case in a simple and digital way to colleagues [1].

### 2.10. Sequential Search Strategy

After the first literature analysis, all of the article titles were screened so as to exclude irrelevant publications, case reports, and non-English publications. Then, researches were not selected based on the data obtained from screening the abstracts. The final stage of screening involved reading the full texts in order to confirm each study’s eligibility, based on the inclusion and exclusion criteria.

### 2.11. Data Extraction

The data were independently extracted from the studies in the form of variables, according to the aims and themes of the present review, as listed onwards.

### 2.12. Data Collections

The data were collected from the included articles, and were arranged in the following fields (Table 1):

“Author (Year)”—revealed the author and year of publication
“Dental Field”—the dental field of Digital Smile Design was used

### 2.13. Risk of Bias Assessment

Two authors undertook the assessment of risk of bias during the data extraction process. For the included studies, this was conducted using the Cochrane Collaboration’s two-part tool for assessing the risk of bias [7,8]. An overall risk of bias was then assigned to each trial, according to Higgins et al. [8]. The levels of bias were classified as follows: low risk, if all of the criteria were met; moderate risk, when only one criterion was missing; high risk, if two or more criteria were missing; and unclear risk, if there were too few details to make a judgement about the certain risk assessment.

## 3. Results

The results were collected from all of the articles that were taken into consideration. The articles that talk about Digital Smile Design and its use in the field of rehabilitative and restorative dentistry were used. In the article, we have not only taken into consideration the “communicative” utility of the software towards the patients, but also that of therapeutic planning and of aesthetic and functional rehabilitation. The articles included in our review already provide important information regarding the field of use of the current digital techniques. Surely, in the first place, the most common field of use is prosthetic and dental restoration. In second place are the positions that mention digital techniques for periodontal purposes instead. Later, we will review these works more closely. Although these techniques are modern and relatively new, the purpose of this work is not to indicate whether these techniques are reliable or not, because the available data available are still few. The aim is to highlight the use-trend in different dental fields.

### 3.1. Study Selection

**Figure 1:**
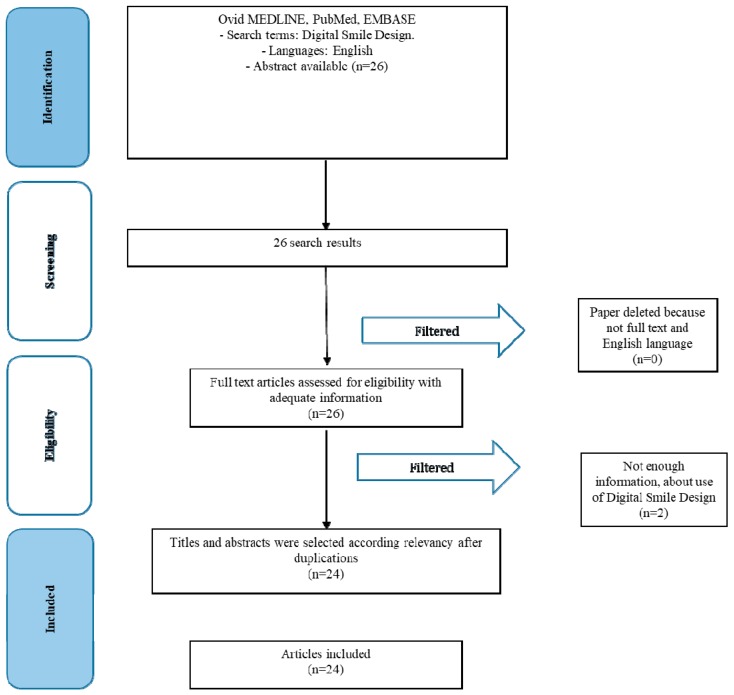
PRISMA flow diagram.

The article review and data extraction were performed according to the Preferred Reporting Items for Systematic Reviews and Metanalyses PRISMA flow diagram (Figure 1). The initial electronic and hand searches retrieved 26 articles. After the titles and abstracts were reviewed, only 24 articles were included.

### 3.2. Study Characteristics

During the selection of the studies, their individual characteristics were evaluated. The characteristics assessed mainly concerned the field of use of Digital Smile Design (Table 1), as follows:

- Restorative dentistry
- Periodontal surgery
- Implantology
- Guided bone regeneration
- Orthodontics
- Maxillofacial surgery

**Table 1:** Digital Smile Design use.

Author	Year of Publications	Field of Digital Smile Design Use					
		Restorative and Prosthodontics	Periodontal Surgery	Implantology	Guided Bone Regeneration	Orthodontics	Maxillofacial Surgery
Santos et al. [9]	2017		✔				
Meereis et al. [10]	2016	✔					
Cattoni et al. [11]	2016	✔					
Omar et al. [12]	2018						
Arias et al. [13]	2015		✔				
Perez-Davidi [14]	2015	✔					
Tak On et al. [15]	2016						
Daher et al. [16]	2018						
Trushkowsky et al. [17]	2016	✔					
Garcia et al. [18]	2018	✔					
Coachman et al. [19]	2017	✔		✔			
McLaren et al. [20]	2018						
Stanley et al. [21]	2018	✔					
Rojas-Vizcaya et al. [22]	2017	✔		✔	✔		
Pinzan-Vercelino et al. [23]	2017	✔				✔	
Marsango et al. [24]	2014	✔	✔			✔	
Frizzera et al. [25]	2017	✔	✔				
Veneziani [26]	2017	✔	✔		✔	✔	
Pimentel et al. [27]	2015	✔					
Paredes-Gallardo et al. [28]	2017						✔
Halley [29]	2015						
Coachman et al. [30]	2016	✔					
Miranda et al. [31]	2016	✔					

✔: Selected papers after the inclusion criteria.

### 3.3. Risk of Bias within Studies

Summarizing the risk of bias for each study, most of the studies were classified as unclear risk. More studies were considered as having a low risk of bias.

### 3.4. Risk of Bias across Studies

There were several limitations present in the current review. The current review includes studies written in English only, which could introduce a publication bias. There were various degrees of heterogeneity in each study design, case selection, and treatment provided among the studies.

## 4. Discussion

Today, dental care tends to be more conservative than in the past, above all thanks to the advances in medicine. In this section, we want to examine more closely the results obtained by the individual articles evaluated. According to Santos et al., the use of dental planning software can also be used for periodontal surgery. In their article, they considered a case of periodontal plastic surgery appropriately programmed through the Digital Smile Design. Digital Smile Design (DSD) allows for a complete planning of the treatment; the programmed results and those obtained after the surgery are comparable. The increase in the clinical crown is an intervention that must always be appropriately planned. Patients accept better oral surgical techniques if techniques such as DSD are used [9]. In another study, Meereis et al. considered Digital Smile Design for aesthetic rehabilitation, and the usefulness of this tool is again confirmed. In this work, it is considered with the combined approach of gingival plastic surgery and restorative dentistry. In this case, the patient’s rehabilitation is performed with lithium disilicate glass ceramic veneers [10]. In the work of Cattoni et al., prosthetic planning is carried out with a digital workflow. The limit of these technologies, according to the authors, is represented by a two-dimensional (2D) workflow—the technique faced by the authors in this case provides a totally digital CAD/CAM process to minimize errors. The three-dimensional (3D) planning is sent to the dental laboratory for the fabrication of the prosthetic products. The technique of combining the Digital Smile Design digital workflow with the .stl files from the digital optical impression allows for the realization of these artefacts in the laboratory [11]. A study by Omar and Duarte published in the Saudi Dentistry journal in 2018 reviewed various programs used for Digital Smile Design, and therefore for treatment planning. In this study, different programs were evaluated (Photoshop CS6, Keynote, Planmeca Romexis Smile Design, CEREC SW 4.2, Aesthetic Digital Smile Design, Smile Designer Pro, DSD App, and VisagiSMile). The authors evaluated the reliability of the latter, although some of the programs were not designed for the dental field. The authors in fact focus on this. The possibility of having functions concerning oral structures, such as teeth or gums, makes the work much quicker and more predictable [12]. In 2015, Arias et al. carried out a further study on the approach using Digital Smile, to perform and plan a periodontal surgery in order to solve a gummy smile [13]. Perez-Davidi carried out a study on prosthetic rehabilitations in monolithic material, with CEREC CAD/CAM systems. There is the possibility, therefore, to perform an immediate mock up and then combine the data from Digital Smile Design and CEREC SW4 for manufacturing [14]. The approach to improve patient aesthetics through Digital Smile Design techniques is further confirmed by other works, such as that by Tak On et al. [15]. The authors confirm the possibility of planning the prosthetic treatment in a preventive manner. Some authors, such as Daher et al., have evaluated the possibility of using cheaper strategies to perform an analysis of Digital Smile Design, such as obtaining images with mobile phones [16]. Trushkowsky et al. [17] confirm the possibility of carrying out aesthetic evaluations for oral rehabilitations. According to Garcia et al., the use of new digital tools offers important perspectives for the daily clinic; in his study, a prosthetic rehabilitation of the anterior maxillary area is evaluated, all planned through Digital Smile Design. According to the authors, in addition to offering a powerful tool to propose treatment plans to patients, by showing them, it is also useful for planning [18]. Coachman et al. also evaluated the use of this software for the planning of total rehabilitations. In this study, the software was used to plan a computer-guided surgery, and a computer-aided design and computer-aided manufacturing (CAD/CAM) of the final prosthetic devices. In this case, therefore, Digital Smile Design was useful for the implant-prosthetic rehabilitation [19]. According to a study by McLaren et al., cosmetic and aesthetic dentistry have undergone a new push from these digital tools. The authors also show how software like Adobe Photoshop can highlight alterations to the smile and can be useful in the analysis of the patient [20]. In 2008, Stanley et al. published an article about digital workflow. The patient of his work, suffering from a Temporo-Mandibular Joint TMJ disorder, underwent a prosthetic rehabilitation with veneers and crowns, with a minimally invasive approach, in order to rehabilitate the loss of vertical dimension and to resolve his joint pains. The protocol was completely digital, using Digital Smile Design for planning and the CAD/CAM techniques for production [21]. An article in the Journal of Prosthetic Dentistry speaks about the possibility of performing a total rehabilitation supported by implants and a guided bone rehabilitation based on Digital Smile Design. The patient suffering from a serious bone deficiency, after a digital planning, therefore, is subjected to a vertical and horizontal bone augmentation. For this reason, surgical templates have been built, and then the fixtures are positioned in order to allow for implant-prosthetic rehabilitation. The final rehabilitation involves a total prosthesis screwed on the implants [22]. Pinzan-Vercelino et al. in their article talk about a multidisciplinary approach using Digital Smile Design for aesthetic rehabilitations in patients with medial diastema of the maxilla [23]. Marsango et al., in Oral and Implantology, talk about the digital workflow. The aesthetic planning and simulation of the dental treatment were carried out on two arches, and subsequently, the scans were sent to a laboratory for production using CAD/CAM techniques. In this case, a prosthetic implant prosthetic rehabilitation was evaluated [24]. A further work taken into consideration sees the use of Digital Smile Design for different dental fields. These techniques, according to the authors, can be used for periodontology, implantology, and prosthetics. Thanks to this software, it is also possible to program pre-prosthetic surgery in detail [25]. Veneziani M. proposed a study on this approach using Digital Smile Design for the treatment of complex cases. In this article, he evaluated the possibility of rehabilitation with veneers, including different branches of dentistry such as periodontal therapy, mucogingival, restorative dentistry, orthodontics, and prosthetics. Therefore, a multidisciplinary approach for the patient thanks to this digital protocol using still porcelain veneers was found [26,27]. Another interesting article talks about the possibility of rehabilitating a second-class brachyphosis patient with a mandibular asymmetry. This complex case involves planning with Digital Smile Design. The multidisciplinary approach in this case includes periodontal, oral, orthodontic, prosthetic, and maxillofacial surgery. The surgical treatment consisted of an osteotomy of the bilateral mandible and a genoplasty; after the surgery, the plates were screwed, and finally removed. Orthodontic treatment followed the surgery and the prosthetic rehabilitation was scheduled [28]. Elaine Halley spoke about the future of dentistry and 3D planning in changing the facial appearance of patients [29]. The latest article to be taken into consideration in this review, in addition to evaluating the use of Digital Smile Design in aesthetic dentistry, sees a case of rehabilitation of the anterior jaw, in this case using metal-free ceramic crowns, passing first for the provisional function of conditioning the gingival tissues [30,31]. In this work, we therefore broadly considered the use of a digital workflow in order to allow for an oral rehabilitation in the different fields of dentistry. Certainly, this technique, already mentioned by some authors [22], is also useful for correcting important bone defects after invasive surgeries in the case of new cancers. Digital techniques can program correct alveolar preservation after extractions with the different techniques present [32]. Soft tissue management and proper planning allow for gingival health to be maintained in our patients [33,34]. In addition, minimally invasive rehabilitations that can be designed allow for the maintenance of dental tissues, while ensuring a correct interface between the dental and prosthetic surfaces, so that there may be correct adhesion [35]. Surely, these types of techniques will progressively tend to replace all of the analog techniques, such as impression techniques, which have different disadvantages [36]. Indeed, with the possibility of being able to provide rehabilitation, it will be possible to make this more predictable. The ideal situation would be to have the advantages from the digital evolution in all of the fields of dentistry—imagine the possibility of knowing precisely the margins of a prosthesis, or even the root canal anatomy, for prosthetic rehabilitation, all the way through to the pins. In this way, it would be possible to predict the angulation and orientation of these beforehand, so as to program the prosthesis, or, for example, by knowing the canal anatomy [37], to know how our instruments will behave during the different therapies [38]. The possibility, in fact, to be able to predict the behavior of the tools that are used by the clinician, would be a very useful target, especially in the aforementioned therapies, where the tools can affect a result if they go against fracture, or experience breakage or wear during treatment. Some materials also suffer, as a result of mechanical fatigue, or even physical or chemical treatments [39]. The latter can also occur within the oral cavity itself [40]. With the improvements in the software over the next few years, it will be possible to program the rehabilitation of a patient by combining the files coming from a CT scan or a Cone Beam, along with the .stl files of an oral impression or a facial scan and a photo. All of this guarantees the rehabilitation desired by the patients as well as guaranteeing their satisfaction [41]. Combining all of this with the predictable wear and tear of different materials, would definitely make rehabilitation more reliable. At this stage, this is the main limitation. Several treatment opportunities, in the field of oral surgery and prosthodontics, as well as dental materials, are available today. Future perspective studies should be directed to managing those different anatomical areas related to the different disciplines. However, the results of the present study still underline how, even if there is significant progress in the field of computer-assisted medicine and dentistry, the clinical evaluation of the patients during the first visit, and therefore the close cooperation between the oral surgeons, radiologists, and prosthodontics, cannot be replaced without compromising the final long term aesthetic and functional results of the patients involved in the treatment. Further clinical studies will help to improve on the management of difficult cases.

Surely, this analysis of the individual articles included in the review has brought to light other evidence regarding the use of digital techniques for dental or medical planning. The fields of use have been clarified, how these techniques are used and what reliability they possess have also been clarified, although it is not possible to obtain statistical results as a result of a lack of data.

### Limitations

This work takes into consideration the fields of use of Digital Smile Design, so it does not compare the statistical data from the individual studies. 

The low number of studies in the literature for this topic unfortunately represent a disadvantage. This is a very current topic and is still not widely dealt with in the scientific field, and our study clearly explains what the fields of use are in dentistry for using this digital instrument, so it is anticipated to have good scientific confirmation. Having a large number of scientific articles available on this topic that contain detailed information on the reliability, accuracy, and predictability of these methods, would certainly be a good starting point for further review.

## 5. Conclusions

In could be concluded from all of the articles present in the literature regarding Digital Smile Design, that this tool provides important information to the clinician and patient. Patients can view their rehabilitations even before they start, and this can have important medico-legal functions. In recent years, these digital techniques have undergone a great positive evolution. It is also possible to remember that other techniques, such as engineering finite element analysis, have provided great support to the biomedical field, allowing for the simulation of structures even before being tested on patients, improving the quality of the rehabilitations and the predictability of the latter. With regard to planning, digital instruments appropriately interfaced with other digital files concerning radiographs and dental laboratory machines thus allow for rehabilitations that are more predictable. Indeed, technology has been evolving in this field in recent years, and will continue to include big updates on Digital Smile Design. However, facial scans would be able to make predictions of bone growth in children, to plan orthodontic–orthopedic rehabilitations, and then drive the proper growth of the jaws.
